# The ProCaSP study: quality of life outcomes of prostate cancer patients after radiotherapy or radical prostatectomy in a cohort study

**DOI:** 10.1186/s12894-015-0025-6

**Published:** 2015-04-10

**Authors:** Nora Eisemann, Sandra Nolte, Maike Schnoor, Alexander Katalinic, Volker Rohde, Annika Waldmann

**Affiliations:** Institute of Cancer Epidemiology, University of Luebeck, Ratzeburger Allee 160, 23562 Luebeck, Germany; Medical Clinic, Department of Psychosomatic Medicine, Charité - Universitätsmedizin Berlin, Charitéplatz 1, 10117 Berlin, Germany; Deakin University, 221 Burwood Highway, Burwood, VIC 3125 Australia; Institute of Social Medicine and Epidemiology, University Hospital Schleswig-Holstein, Campus Luebeck, Ratzeburger Allee 160, 23562 Luebeck, Germany; Medical Practice of Urology, Auguststr. 4, 23611 Bad Schwartau, Germany; Department of Urology, Pediatric Urology and Andrology, Justus Liebig University of Giessen, Rudolf-Buchheim-Str. 7, 35392 Giessen, Germany

**Keywords:** Prostatic neoplasms, Radiotherapy, Prostatectomy, Quality of life, Cohort study

## Abstract

**Background:**

This study describes and compares health-related quality of life (HRQOL) of prostate cancer patients who received either radical prostatectomy (nerve-sparing, nsRP, or non-nerve-sparing, nnsRP) or radiotherapy (external RT, brachytherapy, or both combined) for treatment of localised prostate cancer.

**Methods:**

The prospective, multicenter cohort study included 529 patients. Questionnaires included the IIEF, QLQ-C30, and PORPUS-P. Data were collected before (baseline), three, six, twelve, and twenty-four months after treatment. Differences between groups’ baseline characteristics were assessed; changes over time were analysed with generalised estimating equations (GEE). Missing values were treated with multiple imputation. Further, scores at baseline and end of follow-up were compared to German reference data.

**Results:**

The typical time trend was a decrease of average HRQOL three months after treatment followed by (partial) recovery. RP patients experienced considerable impairment in sexual functioning. The covariate-adjusted GEE identified a significant - but not clinically relevant - treatment effect for diarrhoea (b = 7.0 for RT, p = 0.006) and PORPUS-P (b = 2.3 for nsRP, b = 2.2 for RT, p = 0.045) compared to the reference nnsRP. Most of the HRQOL scores were comparable to German norm values.

**Conclusions:**

Findings from previous research were reproduced in a specific setting of a patient cohort in the German health care system. According to the principle of evidence-based medicine, this strengthens the messages regarding treatment in prostate cancer and its impacts on patients’ health-related quality of life. After adjustment for baseline HRQOL and other covariates, RT patients reported increased symptoms of diarrhoea, and nnsRP patients decreased prostate-specific HRQOL. RP patients experienced considerable impairment in sexual functioning. These differences should be taken into account by physicians when choosing the best therapy for a patient.

**Electronic supplementary material:**

The online version of this article (doi:10.1186/s12894-015-0025-6) contains supplementary material, which is available to authorized users.

## Background

Prostate cancer has a very long period of latency of up to 15–20 years during which the disease is histologically present but has not yet become symptomatic. Autopsy studies have shown that a relevant proportion of men – depending on age and on ethnicity up to 83% (US whites, age group 71–80 years) – has an occult prostate cancer [1,2].

Despite the conflicting evidence of benefits and harms of PSA screening [3,4] and no coverage of costs by statutory health insurance in Germany, testing on patient request is common practice (approx. 30% per year in men aged 45+) [5]. With the increasing use of PSA testing it can be assumed that nowadays a relevant proportion of former asymptomatic, occult cancer will be diagnosed. Today, prostate cancer is the most common malignancy in men in Germany with about 65,000 incident cases each year and a five-year prevalence of about 280,000 men [6].

As every screening test, including the PSA test, aims to detect occult cancers at an early stage, one can assume that a relevant proportion of screening detected prostate cancers would not have become symptomatic during life time and thus can be regarded as overdiagnosis. In these patients cancer treatment may have no benefit but may result in treatment associated morbidity [7]. As there is a moral imperative for treatment of cancer patients independent of tumour size, most young to middle-aged patients with non-metastatic prostate cancer receive some kind of invasive treatment. The main therapeutic strategies are radical prostatectomy (RP), external radiation, and interstitial brachytherapy. Each of above therapies can achieve a five-year cancer-specific survival of more than 90% [8]. Because of the favourable prognosis of early stage tumours and because of treatment morbidity, outcomes other than ‘survival’ are increasingly important [9].

As a result, many studies focus on middle-term or long-term health-related quality of life (HRQOL) outcomes. For example, non-nerve-sparing RP (nnsRP) has been shown to lead to a higher rate of incontinence and impotence. Nerve-sparing RP (nsRP) has been reported to have a positive impact on postoperative incontinence and impotence [10,11], and is preferred whenever possible. Examples of side effects of radiotherapy (RT) include irritable urinary and bowel problems, i.e. symptoms that can fundamentally compromise patients’ overall well-being [12-14]. While it is desirable to detect early stages of prostate cancer and thus lower mortality rates of prostate cancer, these therapies can have substantial impact on the HRQOL of cancer patients.

In view of the high proportion of overdiagnosis in prostate cancer [15-17] and while there is ‘no optimal way to treat localised prostate cancer’ [18], the pros and cons of the available treatment options must be considered. The ProCaSP Study was an observational study aimed at comparing longitudinal HRQOL outcomes across a range of treatment groups for localised prostate cancer in real-world treatment situations. In detail, it was aimed at exploring inter-group differences between two prostatectomy and three radiotherapy groups: 1) nerve-sparing RP, 2) non-nerve-sparing RP, 3) brachytherapy (brachyRT), 4) external RT (externRT), and 5) combined external and brachytherapy (combRT). In addition, HRQOL of the cancer patients was compared to a reference population.

## Methods

### The ProCaSP Study

The Prostate Cancer, Sexuality, and Partnership (ProCaSP) Study was a German prospective multicenter study. ProCaSP was aimed at evaluating HRQOL outcomes of patients with localised prostate cancer treated with either radical prostatectomy or radiotherapy. Furthermore, the data included patients’ perceptions on sexuality and partnership (data not reported) and their partners’ HRQOL [19]. Inclusion criteria were stages T1a to T3b according to the TNM-classification 5th edition [20], no transurethral prostate resection within the last six months, and prostate volume ≤50 ml. Exclusion criteria were positive skeletal scintigraphy, synchronic or metachronic secondary tumours, participation in another study, and insufficient capacity to contract. Patients were classified into different risk groups [21].

The choice of treatment was based on a shared decision between patient and urologist. The decision regarding nerve-sparing versus non-nerve-sparing procedure was made by the hospital surgeon during surgery.

### Data collection

From 2002 to 2006, patients were recruited in ten German study locations. Follow-up was completed in 2008. Data were collected before (baseline), three, six, twelve, and twenty-four months after the start of treatment. Patients were asked to provide information on sociodemographic characteristics, treatment, sexual functioning, and HRQOL.

Cancer-specific HRQOL, prostate-specific HRQOL, and sexual functioning were measured by validated questionnaires (see below). Urinary functioning, which is of interest as it is often compromised after RT, was measured by the best available instrument in German language at that time, the Prostate Specific Module (PSM) [22]. However, in our study cohort the PSM was found to have insufficient psychometric properties for some of the PSM scales and the data was, therefore, not considered in this analysis.

### European organisation of research and treatment in cancer quality of life questionnaire, core module (EORTC QLQ-C30)

The QLQ-C30 is a cancer-specific HRQOL measure. Version 3.0 comprises 30 items covering five functioning scales, three symptom scales, six symptom items, and two items on global HRQOL. Raw scores can be transformed to a range between 0 and 100, with higher functioning scores representing better functioning and higher scores for symptoms/problems representing worse conditions, respectively [23]. The minimal clinically important difference (MCID) was set at ten points [24].

### Patient-oriented prostate utility scale (PORPUS)

The PORPUS questionnaire is a prostate-specific HRQOL instrument. The single HRQOL score, the PORPUS-P [25,26], ranges from 0 to 100, with higher values representing higher HRQOL. Differences of five points were interpreted as the MCID [25].

### International index of erectile function (IIEF)

The IIEF-15 measures sexual functioning. The 15 items were summed up to a total score ranging between 5 and 75. Higher values correspond to higher functioning.

### Ethics and consent

The study protocol was approved by the ethics committee of the Giessen University Hospital, Germany. All patients provided written informed consent.

### Statistical analysis

Analyses were performed using R 3.0.2 [27]. First, patients’ sociodemographic characteristics and their tumor-specific data were described with means (standard deviations), absolute and relative frequencies. Differences in patients’ characteristics between the three main groups (nnsRP, nsRP, RT) were tested with Chi-square and F-tests, respectively. Statistical significance was defined as p< =0.05.

Second, time trends of mean HRQOL were presented graphically by main treatment groups (nnsRP, nsRP, RT) and by RT subgroups (brachyRT, externRT, and combRT). Although we did not consider sexual functioning as a main outcome, changes over time are shown to illustrate the different trends of the treatment group.

Third, the relationship between treatment (nnsRP, nsRP, RT) and HRQOL was analysed using generalised estimating equations (GEE) that account for the correlation between repeated HRQOL observations. The HRQOL observations of the follow-up period were modeled depending on respective treatment option, while adjusting for baseline characteristics (baseline HRQOL, age, having a partner (yes/no), highest education level (no graduation, 8–9 years (‘Hauptschulabschluss’), 10–11 years (‘Realschulabschluss’), >= 12 years high school (‘(Fach-)Abitur’), working (yes/no), residence (rural/urban), tumour stage (T-category of the TNM-classification), pre-therapeutic Gleason score, pre-therapeutic PSA score, and sexual functioning at baseline (IIEF total scale)). Regression coefficients of treatment options with their confidence intervals and Wald tests for testing the effect of treatment option are reported. The analysis was repeated after splitting the RT group into the three subgroups brachyRT, externRT, and combRT.

Most HRQOL domains and some patient characteristics were affected by missing values ranging from 0% to 41.1%, with 22.7% of all observations missing. Multiple imputation is known to be a statistically sound method for handling incomplete data [28]. Hence, missing values were imputed ten times depending on all patient characteristics and on those observation times of HRQOL domains with a correlation of at least 0.5. Results were pooled according to Rubin’s Rule [28].

Fourth, the HRQOL scores at baseline and twenty-four months after treatment of the three main treatment groups as well as the three RT subgroups were compared to German norm values for the EORTC QLQ-C30 [29] by calculating the reference score in a population with a similar age distribution and presenting the difference between the treatment groups’ score and the reference value.

### Post hoc statistical power analysis

A post hoc power analysis was conducted using the software package G*Power3 [30] with α = 0.05, two-tailed. The calculation was based on a repeated measure MANOVA for the two groups with the highest and the lowest HRQOL, which had also the lowest sample sizes: nsRP (n = 127) and RP (n = 133). The expected treatment difference was the MCID, namely 10 for HRQOL and 5 for PORPUS-P. The standard deviation (SD) (between 10 and 20 for the different outcomes) and the correlation between the four repeated measures of the individuals (between 0.5 and 0.8) were estimated from the data. Power was generally far above 95%.

## Results

### Patients and tumour characteristics

516 of the initial 529 patients had complete or partially complete HRQOL data and were included. Approximately one of five observations for each HRQOL outcome (23.9%) and one of the ten baseline covariate values (13.3%) were missing for every patient. 256 patients received nnsRP, 127 nsRP, and 133 patients received RT, of which 44 were treated by brachyRT, 52 by externRT, and 37 by combRT. More than half of those patients treated with RT, where information on pre-baseline androgen deprivation therapy was available, additionally received androgen deprivation therapy (ADT) (52%).

Compared to patients receiving other treatment options, patients who underwent nsRP surgery were younger, were more often employed, more often living in a rural area, and had a better baseline sexual functioning (Table 1). Tumours that could not be treated nerve-sparingly during surgery had more often an advanced stage. RT patients were on average older than patients in the other treatment groups, more often not employed, living in urban areas, had a higher PSA level, more often a small tumour stage, and a lower sexual functioning. Comparison of D’Amico risk stratification revealed a much lower risk for the RT treatment groups. More than 80% of the nnsRP and nsRP patients were considered at high risk compared to less than 40% of the RT patients (data after multiple imputation, not shown). Pre-treatment PSA levels were highest in the combRT group and lowest in the nsRP group, while Gleason levels were highest in the nnsRP group and lowest in the brachyRT group.

**Table 1:** **Sociodemographic characteristics and tumor specific data**

	**Radical prostatectomy**		**Radiotherapy**				**p-value** ^**1**^
	**Non-nerve-sparing (reference)**	**Nerve-sparing**	**Total**	**Brachytherapy**	**Combined (External/brachytherapy)**	**External**	
	**(n = 256)**	**(n = 127)**	**(n = 133)**	**(n = 44)**	**(n = 37)**	**(n = 52)**	
Age [mean ± SD]	64.2 ± 6.3	59.7 ± 6.1	66.5 ± 5.5	65.0 ± 6.0	66.8 ± 5.5	67.7 ± 4.8	<0.001
Partnership [N (%)]							
Not living with a partner	14 (5.5)	6 (4.7)	10 (7.5)	4 (9.1)	3 (8.1)	3 (5.8)	0.586
Married or having a spouse	239 (93.4)	120(94.5)	121 (91.0)	39 (88.6)	33 (89.2)	49 (94.2)	
Missing	3 (1.2)	1 (0.8)	2 (1.5)	1 (2.3)	1 (2.7)	0 (0.0)	
Education [N (%)]							
without	-	-	2 (1.5)	0 (0.0)	0 (0.0)	2 (3.8)	0.113
8-9 years	127 (49.6)	50 (39.4)	65 (48.9)	20 (45.5)	21 (56.8)	24 (46.2)	
10-11 years	59 (23.0)	31 (24.4)	27 (20.3)	11 (25.0)	10 (27.0)	6 (11.5)	
>=12 years	70 (27.3)	45 (35.4)	38 (28.6)	13 (29.5)	6 (16.2)	19 (36.5)	
Missing	-	1 (0.8)	1 (0.8)	0 (0.0)	0 (0.0)	1 (1.9)	
Employment status [N (%)]							
Employed	70 (27.3)	67 (52.8)	17 (12.8)	9 (20.5)	2 (5.4)	6 (11.5)	<0.001
Non employed	180 (70.3)	59 (46.5)	114 (85.7)	33 (75.0)	35 (94.6)	46 (88.5)	
Missing	6 (2.3)	1 (0.8)	2 (1.5)	2 (4.5)	0 (0.0)	0 (0.0)	
Living area [N (%)]							
Rural	149 (58.3)	85 (66.9)	56 (42.1)	29 (65.9)	18 (48.6)	9 (17.3)	<0.001
Urban	102 (39.8)	40 (31.5)	71 (53.4)	12 (27.3)	17 (45.9)	42 (80.8)	
Missing	5 (2.0)	2 (1.6)	6 (4.5)	3 (6.8)	2 (5.4)	1 (1.9)	
Pre-therapeutic Gleason score, [mean ± SD]	6.6 ± 1.2	6.5 ± 1.0	6.0 ± 13.1	5.4 ± 1.4	6.1 ± 1.6	6.4 ± 1.1	0.110
Missing [N]	2	1	33	25	7	3	
Pre-therapeutic PSA score, [mean ± SD]	11.4 ± 21.3	8.0 ± 4.7	13.7 ± 17.0	10.8 ± 15.0	17.6 ± 14.4	13.2 ± 19.9	0.032
Missing [N]	5	1	2	1	1	-	
Pre-baseline androgen deprivation therapy [N (%)]							
Yes	10 (3.9)	1 (0.8)	51 (38.3)	20 (45.4)	13 (35.1)	18 (34.6)	<0.001
No	185 (72.2)	81 (63.8)	46 (34.6)	16 (36.4)	12 (32.4)	19 (36.5)	
Missing	61 (23.8)	45 (35.4)	36 (27.0)	8 (18.2)	12 (32.4)	16 (30.8)	
Tumour stage at diagnosis [N %)]							
T1	2 (0.8)	-	32 (24.0)	1 (2.3)	1 (2.7)	29 (55.8)	<0.001
T2	142 (55.5)	103 (81.1)	39 (29.3)	8 (18.2)	17 (45.9)	14 (26.9)	
T3	105 (41.0)	21 (16.5)	20 (15.0)	1 (2.3)	12 (32.4)	7 (13.5)	
Missing	7 (2.7)	3 (2.4)	43 (32.3)	34 (77.3)	7 (18.9)	2 (3.8)	
Risk stratification [N (%)]							
Low	13 (5.1)	7 (5.5)	25 (18.8)	6 (13.6)	0 (0.0)	19 (36.5)	<0.001
Intermediate	33 (12.9)	15 (11.8)	22 (16.5)	2 (4.5)	8 (21.6)	12 (23.1)	
High	205 (80.1)	103 (81.1)	47 (35.3)	4 (9.1)	25 (67.6)	18 (34.6)	
Missing	5 (1.9)	2 (1.6)	39 (29.3)	32 (72.7)	4 (10.8)	3 (5.8)	
Operation condition [N (%)]							
Retropubic	251 (98.0)	124 (97.6)	n.a.	n.a.	n.a.	n.a.	n.a.
Perineal	5 (2.0)	2 (1.6)					
Missing	-	1 (0.8)					
Sexual functioning, [mean ± SD]	41.5 ± 21.2	54.7 ± 16.5	33.1 ± 22.3	26.7 ± 22.1	32.4 ± 21.6	39.7 ± 21.7	<0.001
Missing [N]	92	36	44	11	15	18	

^1^Chi-square and F- test for the three groups (non-nerve-sparing radical prostatectomy, nerve-sparing radical prostatectomy, and total radiotherapy), respectively.

### Sexual functioning - IIEF

**Figure 1:**
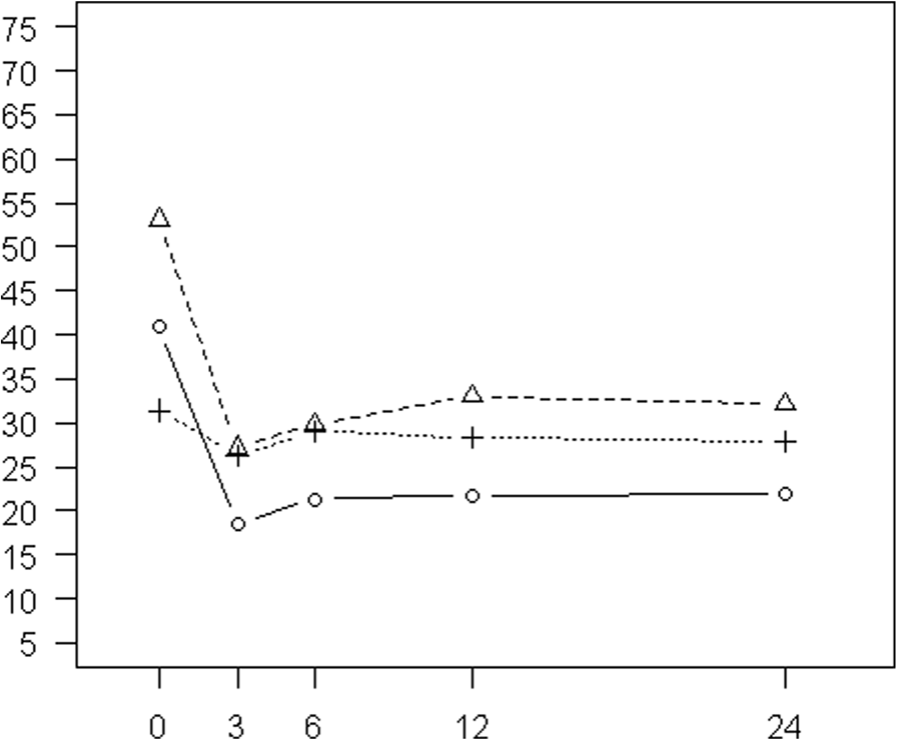
**Sexual functioning (IIEF).** Mean scores of nnsRP, nsRP, and RT cancer patients at baseline and during the 24-month follow-up period after multiple imputation of missing values (solid line: nnsRP, dashed line: nsRP, dotted line: RT).

Figure 1 shows that the average sexual functioning of the RT patients remains on a similar low level over the whole observation period. Patients receiving nnsRP start off with a higher sexual functioning, but end up with the lowest scores. Patients receiving nnsRP have the highest baseline scores, but drop by more than 20 points, ending after a small recovery at a 24-month score similar to the baseline score in RT patients. Additional file 1 presents the trends for the three RT subgroups, which end with very similar scores after 24 months of follow-up.

### Generic HRQOL – QLQ-C30

**Figure 2:**
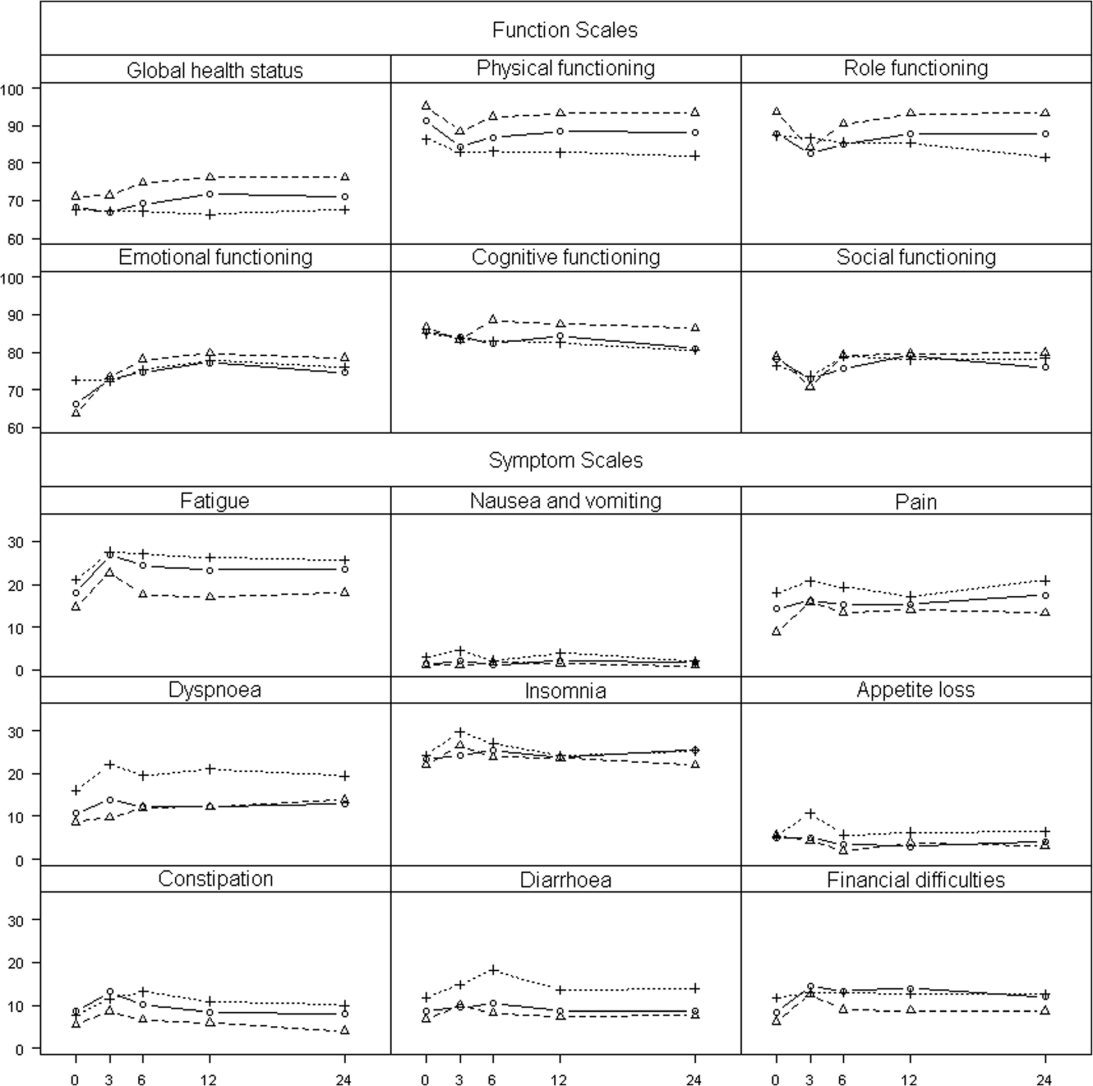
**Health-related QoL (QLQ-C30).** Mean scores of nnsRP, nsRP, and RT cancer patients at baseline and during the 24-month follow-up period after multiple imputation of missing values (solid line: nnsRP, dashed line: nsRP, dotted line: RT).

Several of the baseline functioning or symptom scales differed to a clinically relevant extent across the treatment groups (physical, role, emotional, and social functioning, fatigue, pain, dyspnoea, insomnia, and financial difficulties). In all cases, the RT group – especially the combRT group – had the least favourable baseline values (Figure 2 and Additional file 2). The most favourable value was generally found in the nsRP group, except for emotional functioning, social functioning, and insomnia, which was best in the externRT group. However, both the nsRP and the nnsRP group most often showed higher functioning and fewer symptoms than the total RT group.

Three months after baseline, typically a decrease in functioning scales and an increase in symptom scales were seen, followed by a recovery (Figure 2 and Additional file 2). Due to the smaller number of patients, the time trends of the individual RT groups show a larger variability and the typical time trend can be seen less clearly than in the total RT group. When comparing nnsRP, nsRP, and the total RT group, the following deviations from this pattern were observed: In contrast to the more specific functioning and symptom scales, the global health status was hardly affected by treatment: It remained nearly unchanged for the total RT group but further increased over time for the two RP groups. The increase in emotional functioning was clinically relevant in the nsRP group at six, twelve, and twenty-four months and in the nnsRP group twelve months after baseline. Physical functioning, role functioning, and cognitive functioning worsened over time for the RT group, a group of older patients compared to those in the surgery groups. Among the RT groups, the largest differences were observed for dyspnoea, insomnia, and financial difficulties.

In the multiple regression analysis, only diarrhoea was statistically significantly associated with treatment option after adjusting for baseline HRQOL, age, and other demographical and clinical data (Table 2). RT was estimated to increase the score on the diarrhoea symptom scale by 7 points, while the estimate for nsRP was −0.9. The RT effect was mostly driven by the brachyRT group (effect of 7.8 compared to 4.1 and 4.2 for combRT and externRT). The difference was not clinically relevant.

**Table 2:** **Estimated effect of treatment option on HRQOL in a multiple regression analysis**

	**Main analysis**						**Analysis with separation of different RT treatment groups** ^**1**^						
	**Radical prostatectomy**			**Radiotherapy**		**Wald test**	**Radiotherapy**						**Wald test**
	**Ref.**	**Nerve-sparing**		**Total**			**Brachytherapy**		**Combined (External/brachytherapy)**		**External**		
	**b**	**b**	**95%-CI**	**b**	**95%-CI**	**p-value**	**b**	**95%-CI**	**b**	**95%-CI**	**b**	**95%-CI**	**p-value**
**QLQ-C30**													
*Functioning scales*													
Global health status	0	2.9	(−0.6, 6.4)	−2.0	(−5.9, 2.0)	0.098	−1.0	(−6.2, 4.2)	−2.2	(−8.4, 3.9)	−4.4	(−9.9, 1.2)	0.120
Physical functioning	0	1.7	(−0.6, 4.1)	−0.9	(−4.3, 2.4)	0.198	−0.6	(−5.4, 4.3)	−0.6	(−5.2, 3.9)	−3.5	(−8.9, 2.0)	0.224
Role functioning	0	1.9	(−1.5, 5.3)	−0.5	(−4.8, 3.8)	0.425	−1.6	(−7.9, 4.7)	2.0	(−4.7, 8.7)	−2.5	(−8.8, 3.8)	0.314
Emotional functioning	0	3.8	(−0.2, 7.8)	−2.7	(−7.3, 2.0)	0.074	−2.4	(−8.7, 3.9)	1.1	(−6.2, 8.3)	**−8.6**	**(−15.2, −2.0)**	0.028
Cognitive functioning	0	2.5	(−1.1, 6.2)	−1.8	(−5.5, 1.9)	0.170	−0.7	(−6.0, 4.5)	−2.2	(−8.2, 3.8)	−2.7	(−9.5, 4.0)	0.277
Social functioning	0	1.2	(−3.7, 6.1)	0.1	(−5.4, 5.5)	0.650	−0.7	(−7.9, 6.5)	3.0	(−5.1, 11.1)	−5.7	(−14.3, 3.0)	0.287
*Symptom scales*													
Fatigue	0	−3.6	(−7.8, 0.5)	0.4	(−4.6, 5.3)	0.173	0.1	(−6.6, 6.8)	−1.6	(−9.1, 5.9)	5.4	(−2.0, 12.9)	0.174
Nausea and vomiting	0	−0.5	(−1.6, 0.6)	1.1	(−0.5, 2.7)	0.100	**2.1**	**(0.1, 4.1)**	−0.5	(−2.2, 1.2)	0.6	(−1.8, 2.9)	0.091
Pain	0	0.5	(−3.5, 4.5)	2.7	(−2.2, 7.5)	0.477	3.6	(−3.3, 10.5)	0.1	(−7.2, 7.3)	3.4	(−4.8, 11.6)	0.601
Dyspnoea	0	1.4	(−2.1, 4.8)	3.5	(−0.8, 7.8)	0.177	0.1	(−5.6, 5.8)	6.8	(−0.5, 14.1)	4.8	(−1.5, 11.0	0.122
Insomnia	0	0.1	(−4.7, 5.0)	3.2	(−2.5, 8.9)	0.451	4.3	(−2.9, 11.5)	4.0	(−4.7, 12.7)	6.4	(−2.8, 15.6)	0.291
Appetite loss	0	−0.1	(−2.3, 2.1)	1.5	(−1.7, 4.7)	0.317	1.5	(−1.8, 4.8)	1.0	(−3.7, 5.6)	3.1	(−1.5, 7.6)	0.276
Constipation	0	−1.2	(−5.3, 3.0)	0.4	(−4.1, 4.9)	0.542	1.7	(−4.2, 7.5)	−0.9	(−8.4, 6.5)	3.2	(−5.5, 12.0)	0.362
Diarrhoea	0	−0.9	(−4.4, 2.6)	**7.0**	**(2.5, 11.6)**	**0.006**	**7.8**	**(1.0, 14.5)**	4.1	(−3.3, 11.4)	4.2	(−3.6, 12.0)	**0.048**
Financial difficulties	0	−3.1	(−7.0, 0.8)	−0.2	(−4.7, 4.3)	0.257	−2.7	(−9.1, 3.7)	−0.5	(−8.8, 7.7)	4.9	(−4.0, 13.8)	0.216
**PORPUS**													
PORPUS-P	0	**2.3**	**(0.1, 4.6)**	2.2	(−0.6, 5.0)	**0.045**	2.2	(−1.6, 6.1)	3.3	(−0.6, 7.2)	1.4	(−3.0, 5.7)	0.119

Ref = Non-nerve-sparing radical prostatectomy as reference level.

Bold values indicate significance at the 5%-level.

^1^As the regression coefficients of the radical prostatectomy group did not change much, only the regression coefficients of the radiotherapy subgroups with their confidence intervals and the Wald test p-values are reported.

Both analyses are adjusted for baseline HRQOL, age, having a partner (yes/no), highest education level (no graduation, 8–9 years (‘Hauptschulabschluss’), 10–11 years (‘Realschulabschluss’), >= 12 years high school (‘(Fach-)Abitur’)), working (yes/no), residence (rural/urban), tumour stage (T-category of the TNM-classification), pre-therapeutic Gleason score, pre-therapeutic PSA score, and sexual functioning at baseline (IIEF total scale).

### Disease-specific quality of life – PORPUS-P

**Figure 3:**
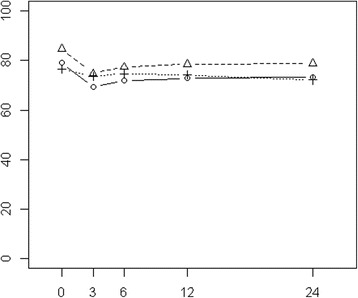
**PORPUS-P.** Mean scores of nnsRP, nsRP, and RT cancer patients at baseline and during the 24-month follow-up period after multiple imputation of missing values (solid line: nnsRP, dashed line: nsRP, dotted line: RT).

At baseline, the PORPUS-P score was highest in nsRP and lowest in RT patients, in particular in patients treated with combRT (Figure 3 and Additional file 3). The difference was clinically relevant. The PORPUS-P score decreased three months after baseline, followed by a partial recovery.

In the covariate-adjusted multiple regression analysis a statistically significant but not clinically relevant association of main treatment option and HRQOL during the follow-up period was observed (Table 2), with nsRP patients having a significantly higher HRQOL and RT patients also having a (not statistically significant) higher HRQOL than nnsRP patients.

### Comparison of generic HRQOL to German norm values

Overall, scores of the treatment groups at baseline and twenty-four months after treatment were mostly comparable to German norm values for the EORTC QLQ-C30 [29] (Table 3). However, patients treated with RP had clinically relevant lower scores of fatigue and pain at baseline, and the low pain score persisted for the nsRP patients for twenty-four months. The nsPR patients had a clinically relevant higher role functioning than expected at baseline and at the end of the follow-up. No clinically relevant deviations from the norm values were observed for the total group of patients treated with RT. However, the brachyRT group had a clinically relevant higher role functioning at baseline than expected. The externRT group had a higher role functioning at baseline as well, and lower symptoms of pain, which slightly decreased to a clinically non-relevant level until twenty-four months later. The combRT group had clinically relevant lower emotional and social functioning and higher insomnia scores at baseline, and less pain but more insomnia than the reference population twenty-four months after baseline.

**Table 3:** **Differences between EORTC QLQ-C30 ProCaSP data and norm data**

	**Baseline**								**24 months after baseline**			
	**Radical prostatectomy**		**Radiotherapy**				**Radical prostatectomy**		**Radiotherapy**			
	**nns**	**ns**	**total**	**brachy**	**comb**	**extern**	**nns**	**ns**	**total**	**brachy**	**comb**	**extern**
**Global quality of life**	2.5	5.3	1.5	4.6	−1.7	1.3	5.3	10.3	2.0	3.8	2.8	0.1
**Functioning scales**												
Physical	7.3	9.6	3.0	3.5	2.3	3.2	5.0	8.5	0.0	1.6	−1.3	−0.5
Role	9.7	**14.0**	3.6	**11.0**	2.3	**12.2**	9.7	**14.1**	4.6	−0.5	5.9	9.6
Emotional	−8.7	−9.3	−3.8	−2.8	**−13.2**	1.5	−1.6	4.4	−0.4	2.1	−2.7	−1.6
Cognitive	1.4	2.1	1.7	3.9	−1.5	2.1	−2.4	2.0	−1.6	0.8	−6.5	−0.2
Social	−3.1	−2.9	−4.9	−6.4	**−13.1**	2.4	−5.2	−1.8	−2.2	−7.5	1.9	1.5
**Symptom scales**												
Fatigue	**−10.0**	**−13.5**	−6.9	−9.9	−0.4	−9.3	−4.5	−9.8	−3.4	−3.5	−1.5	−4.6
Nausea/Vomiting	−1.1	−1.7	0.4	1.8	1.9	−1.6	−0.8	−1.7	−0.6	0.8	−1.3	−1.6
Pain	**−12.9**	**−19.0**	−8.7	−8.3	−7.0	**−10.5**	−9.6	**−14.0**	−6.8	−5.4	**−10.8**	−9.4
**Single items**												
Dyspnoea	−9.3	−9.0	−4.9	−9.6	−1.1	−3.3	−8.1	−4.9	−2.7	−6.5	−0.5	0.7
Insomnia	−2.8	−4.8	−1.2	−4.9	**10.7**	−6.6	0.1	−4.5	0.0	−4.0	**10.6**	−1.1
Appetite loss	−2.5	−2.0	−1.6	−3.5	0.6	−1.3	−2.8	−4.3	−0.5	−2.6	−3.8	3.5
Constipation	2.8	0.3	1.7	3.5	2.6	−0.4	1.9	−1.3	2.8	5.4	0.0	3.6
Diarrhoea	0.5	−2.1	3.6	1.7	8.4	2.1	0.4	−0.9	5.8	7.7	0.4	6.5
Financial difficulties	−6.7	−8.4	−3.1	−4.3	4.5	−7.4	−2.8	−6.1	−2.3	0.0	−4.5	−2.3

Legend: positive differences = higher mean functioning or higher mean symptom score in the prostate cancer patient group than in the norm data, negative differences = lower mean functioning or higher mean symptom score in the prostate cancer patient group than in the norm data.

Bold numbers indicate clinical relevance.

nns = non-nerve-sparing, ns = nerve-sparing, brachy = brachytherapy, extern = extern radiotherapy, comb = extern radiotherapy and brachytherapy.

### Sensitivity analysis – complete case analysis

The complete case analysis was based on 248 to 254 patients, depending on the HRQOL measure. Results were mostly similar to the results after multiple imputation presented above. Global health status, emotional functioning, and appetite loss, but not diarrhoea, were additionally found to be significantly (but not clinically relevantly) related to main treatment option.

## Discussion

This study analysed changes of HRQOL of prostate cancer patients over time, the effect of treatment on HRQOL, and compared HRQOL to that in a German reference population.

### Changes over time

The descriptive comparison of time trends showed that a decreased sexual functioning and limited recovery is more common in RP than in RT. Although the comparison does not convey information about a treatment effect on similar/randomised groups, it describes what happens to men with prostate cancer in actual health care. The finding is in concordance with other publications [31,32]. Further, better outcomes in erectile functioning for nsRP patients compared to patients with non-nerve-sparing procedures were also found in previous studies [10,11,33]. Still, erectile functioning is a concern in RP in general [34-36].

In view of generic HRQOL, only minor changes over time were seen across groups. In several other studies these results have been ascribed to the fact that generic instruments are not able to a) distinguish adequately between highly selected groups [26] and b) adequately measure HRQOL after diagnosis because of response shift bias [36,37]. In this analysis, the application of multiple imputation may have obscured differences; the incorporation of uncertainty due to missing values often results in conservative estimates.

A clinically relevant worsening in PORPUS-P and an increase in emotional functioning at all measurement time points after baseline were observed in the RP group (with exceptions for emotional functioning in the nnsRP group). Differences between RP patients and norm values were close to clinical relevance at baseline (−8.7 and −9.3, respectively) [29]. At the end of the follow-up differences in emotional functioning had largely disappeared, which may be ascribed to adaptation processes [38]. Our findings are in contrast to those of a Canadian study observing clinically relevant decreases of prostate-specific HRQOL after radiotherapy [39]. This is particularly interesting as over 50% of our RT patients, where information about pre-baseline androgen deprivation therapy was available, received ADT, i.e. an adjuvant therapy that negatively impacts erectile functioning, social functioning, and global health/HRQOL [39]. Hence, a clinically relevant decrease of HRQOL scores in our RT patients would have been expected. In a joint analysis of the Canadian data and our data, however, we were able to show that RT patients with ADT indeed scored lower HRQOL than RT patients without ADT [40].

### Treatment effects

When controlling for baseline HRQOL, sexual functioning, and other possible confounders in a GEE model, the treatment effect of nsRP compared to nnsRP was favourable for many HRQOL domains and often unfavourable for RT. The treatment effect was significant for the domain diarrhoea of the HRQOL and for the PORPUS-P. The negative effect of RT on bowel functioning is in agreement with previous research [41,42]. However, treatment effects in our analysis never reached clinical relevance.

The treatment groups differed with respect to their baseline HRQOL, sexual functioning, and sociodemographic and tumour-related characteristics. Baseline HRQOL and sexual functioning was generally highest in patients receiving nsRP and lowest in patients receiving RT, especially in the combRT group. RT patients were older than RP patients, and the combRT group had the highest risk profile. Similar results have been reported in comparable international outcome studies [12,41] which is concordant with recommendations in current therapy guidelines such as the EAU-guideline advising an estimated 10-year survival as a precondition for RP but not for RT [43]. Logistic regression indicated that the significant predictors for a successful nerve-sparing surgery (in contrast to a non-nerve-sparing surgery) were a younger age, a higher Gleason score, and a better sexual functioning of the patient, while predictors for choosing radiotherapy were a better T-category, a worse sexual functioning, and an urban living area. However, there was considerable overlap with regard to these variables between the treatment groups, and the covariate adjustment in the regression analyses allowed to derive meaningful treatment effect estimates despite the group differences.

### Comparison to reference population

Patients treated with RP had clinically relevant lower scores of fatigue and pain and higher role functioning than age-matched German men of the general population. The RT group also had lower fatigue and pain scores than suggested by the reference data but the difference was not clinically relevant (except for the externRT subgroup at baseline and the combRT subgroup after 24 months for pain). It can be suspected that the high proportion of participants reporting depression in the reference population caused artificially high norm values for fatigue and pain and low values for role functioning [29]. Even the ‘low’ symptom values (pain, fatigue) of the cancer patients in our study were higher than those reported by Krahn *et al.* (2009) for Canadian early stage prostate cancer patients [39]. The same applies to the role functioning values in the RP group.

### Strengths and limitations

Important strengths of the ProCaSP Study are the multicenter design, the comparison of HRQOL outcomes in several prostate cancer treatment groups, and the application of suitable statistical methods such as GEEs and adjustments for baseline differences. The main limitation of the study is missing data due to drop-out or incomplete questionnaires, especially for IIEF questions; however, by applying multiple imputation methods we achieved a sample size sufficient for stratification of two RP and three RT groups. The exclusion of urinary measures because of insufficient psychometric properties of the Prostate Specific Module limits the scope of our study. Finally, selection bias caused by an overrepresentation of severe cases in university hospitals cannot be ruled out. Possible confounding was handled by adjustment of covariates in the GEE model, although residual confounding cannot be excluded.

## Conclusions

Our results support the findings from previous research. A fundamental basis of both empirical research and evidence-based medicine is the reproducibility of findings in different settings, health care systems, and different cohorts of patients. In conclusion, RT cancer patients scored lowest on HRQOL at baseline and throughout the study. RP had a larger negative impact on sexual functioning than RT. In patients with similar baseline characteristics and similar baseline HRQOL, treatment by RT increased symptoms of diarrhoea, and nnsRP decreased prostate-specific HRQOL. In view of the relevant proportion of overdiagnosis in prostate cancer and in view of improved survival rates for prostate cancer patients, physicians should inform their patients about these differences in HRQOL outcomes and take them into account when deciding which therapy is best for an individual patient.

## Additional files

Additional file 1:
**Sexual functioning Radiotherapy Subgroups.tiff.** Sexual functioning in the radiotherapy subgroups. Mean scores of brachytherapy, external radiotherapy, and combined radiotherapy cancer patients at baseline and during the 24-month follow-up period after multiple imputation of missing values (circle: brachyRT, triangle: externRT, cross: combRT). Additional file 2:
**QLQ-C30 Radiotherapy Subgroups.tiff.** Health-related quality of life in the radiotherapy subgroups. Mean scores of brachytherapy, external radiotherapy, and combined radiotherapy cancer patients at baseline and during the 24-month follow-up period after multiple imputation of missing values (circle: brachyRT, triangle: externRT, cross: combRT). Additional file 3:
**PorpusP Radiotherapy Subgroups.tiff.** PORPUS-P in the radiotherapy subgroups. Mean scores of brachytherapy, external radiotherapy, and combined radiotherapy cancer patients at baseline and during the 24-month follow-up period after multiple imputation of missing values (circle: brachyRT, triangle: externRT, cross: combRT).

## Abbreviations

brachyRT: Brachytherapy
combRT: Combined extern radiotherapy and brachytherapy
externRT: Extern radiotherapy
HRQOL: Health-related quality of life
nnsRP: Non-nerve-sparing radical prostatectomy
nsRP: Nerve-sparing radical prostatectomy
RP: Radical prostatectomy
RT: Radiotherapy
SD: Standard deviation
