# Supplementary figures and images for: The ProCaSP study: quality of life outcomes of prostate cancer patients after radiotherapy or radical prostatectomy in a cohort study
Source: BMC Urol. 2015 Apr 10;15:28. doi: 10.1186/s12894-015-0025-6 (PMC4404598; doi:10.1186/s12894-015-0025-6)

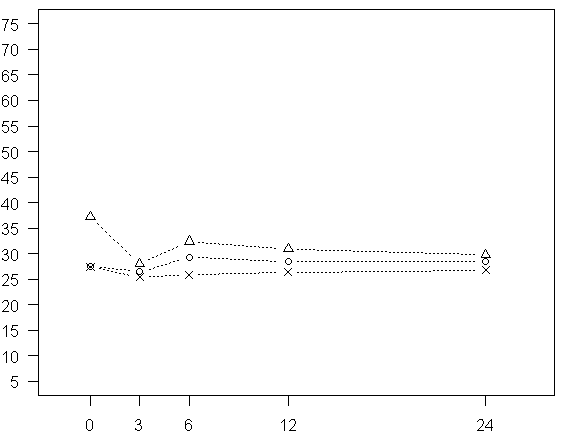

Supplement: Additional file 1: — Sexual functioning Radiotherapy Subgroups.tiff. Sexual functioning in the radiotherapy subgroups. Mean scores of brachytherapy, external radiotherapy, and combined radiotherapy cancer patients at baseline and during the 24-month follow-up period after multiple imputation of missing values (circle: brachyRT, triangle: externRT, cross: combRT). [file 12894_2015_25_MOESM1_ESM.tiff]

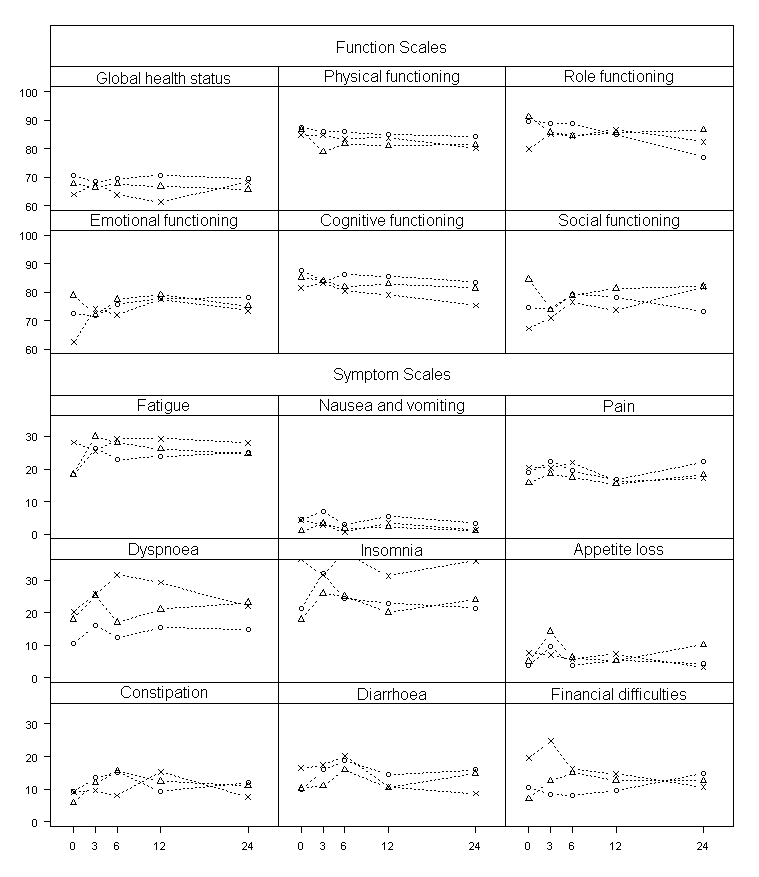

Supplement: Additional file 2: — QLQ-C30 Radiotherapy Subgroups.tiff. Health-related quality of life in the radiotherapy subgroups. Mean scores of brachytherapy, external radiotherapy, and combined radiotherapy cancer patients at baseline and during the 24-month follow-up period after multiple imputation of missing values (circle: brachyRT, triangle: externRT, cross: combRT). [file 12894_2015_25_MOESM2_ESM.tiff]

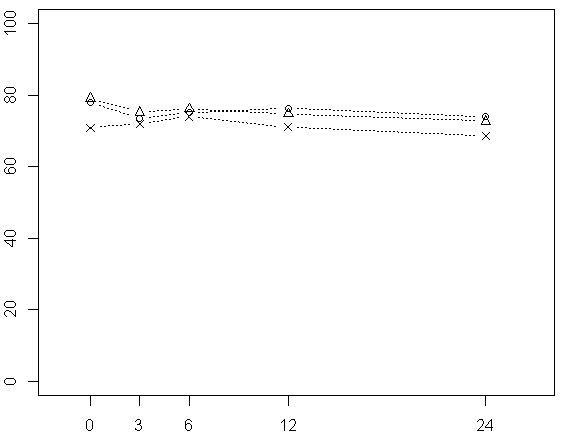

Supplement: Additional file 3: — PorpusP Radiotherapy Subgroups.tiff. PORPUS-P in the radiotherapy subgroups. Mean scores of brachytherapy, external radiotherapy, and combined radiotherapy cancer patients at baseline and during the 24-month follow-up period after multiple imputation of missing values (circle: brachyRT, triangle: externRT, cross: combRT). [file 12894_2015_25_MOESM3_ESM.tiff]
